# 3-Acetyl-5-methyl-1-(4-methyl­phen­yl)-1*H*-pyrazole-4-carboxamide

**DOI:** 10.1107/S1600536810043928

**Published:** 2010-10-31

**Authors:** Hatem A. Abdel-Aziz, Ahmed Bari, Seik Weng Ng

**Affiliations:** a Department of Pharmaceutical Chemistry, College of Pharmacy, King Saud University, Riyadh 11451, Saudi Arabia; b Department of Chemistry, University of Malaya, 50603 Kuala Lumpur, Malaysia

## Abstract

In the title compound, C_14_H_15_N_3_O_2_, the phenyl­ene ring is disordered over two orientations. As a result, the almost planar pyrazole ring (r.m.s. deviation = 0.004 Å) forms dihedral angles of 59.8 (1) and −61.9 (1)° with the two orientations of the phenyl­ene ring. The dihedral angle between the two orientations is 59.2 (1)°. In the crystal, inversion dimers lined by pairs of N—H⋯O hydrogen bonds occur; there is also an intramolecular N—H⋯O bond.

## Related literature

For the synthesis of the title compound, see: Ibrahim *et al.* (1992 ▶).
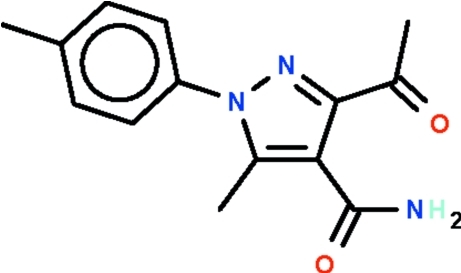


## Experimental

### 

#### Crystal data

C_14_H_15_N_3_O_2_
*M* *_r_* = 257.29
Triclinic, 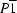

*a* = 5.0521 (6) Å
*b* = 10.4068 (13) Å
*c* = 12.6558 (16) Å
α = 103.295 (2)°
β = 95.338 (2)°
γ = 100.072 (2)°
*V* = 631.39 (13) Å^3^
*Z* = 2
Mo *K*α radiation
μ = 0.09 mm^−1^
*T* = 100 K
0.30 × 0.06 × 0.03 mm

#### Data collection

Bruker SMART APEX diffractometer
6045 measured reflections
2873 independent reflections
2002 reflections with *I* > 2σ(*I*)
*R*_int_ = 0.030

#### Refinement

*R*[*F* ^2^ > 2σ(*F* ^2^)] = 0.050
*wR*(*F* ^2^) = 0.152
*S* = 1.03
2873 reflections
230 parameters
5 restraints
H atoms treated by a mixture of independent and constrained refinement
Δρ_max_ = 0.29 e Å^−3^
Δρ_min_ = −0.34 e Å^−3^

### 

Data collection: *APEX2* (Bruker, 2009 ▶); cell refinement: *SAINT* (Bruker, 2009 ▶); data reduction: *SAINT*; program(s) used to solve structure: *SHELXS97* (Sheldrick, 2008 ▶); program(s) used to refine structure: *SHELXL97* (Sheldrick, 2008 ▶); molecular graphics: *X-SEED* (Barbour, 2001 ▶); software used to prepare material for publication: *publCIF* (Westrip, 2010 ▶).

## Supplementary Material

Crystal structure: contains datablocks global, I. DOI: 10.1107/S1600536810043928/bt5394sup1.cif

Structure factors: contains datablocks I. DOI: 10.1107/S1600536810043928/bt5394Isup2.hkl

Additional supplementary materials:  crystallographic information; 3D view; checkCIF report

## Figures and Tables

**Table 1:** Hydrogen-bond geometry (Å, °)

*D*—H⋯*A*	*D*—H	H⋯*A*	*D*⋯*A*	*D*—H⋯*A*
N3—H31⋯O1	0.88 (3)	1.95 (2)	2.771 (2)	154 (3)
N3—H32⋯O2^i^	0.89 (3)	2.02 (1)	2.906 (3)	177 (3)

Symmetry code: (i) 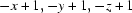.

**Fig. 1.**
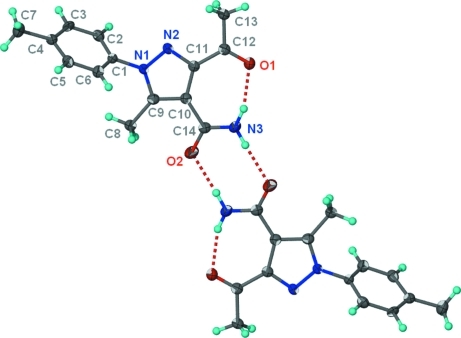
Anisotropic ellipsoid plot (Barbour, 2001) of the title compound showing two molecules related by a center-of-inversion and held together by hydrogen bonds. The probability level is set at 70%; H atoms are drawn as spheres of arbitrary radius, and the disorder is not shown.

## supplementary crystallographic information

### Comment

Pyrazole derivatives have been studied in the context of its biological properties. One class of such compounds is synthesized by reaction of hydrazidolyl chlorides active methylene comounds in basic medium (Ibrahim *et al.*, 1992). The hydrazidolyl chloride in the present study is 2-oxo-*N*'-(4-tolyl)propanehydrazolyl chloride; this reacts with 3-oxobutanamide to yield the title compound (Scheme I). In the title molecule, the phenylene ring adopts two orientations. One orientation has the ring aligned at about 60° and the other has the ring aligned at about -60° with respect to the pyrazoly ring. The two orientations are stagged by another 60°. Two molecules are linked by an N—H···O hydrogen bond about a center-of-inversion to form a dimer. (Fig. 1).

### Experimental

Sodium metal (0.023 g, 1 mmol) was dissolved in absolute ethanol (50 ml); to the solution of sodium ethoxide was added 3-oxobutanamide (0.10 g, 10 mmol). To the clear solution was added 2-oxo-*N*'-(4-tolyl)propanehydrazolyl chloride (0.21 g, 1 mmol). The reaction mixture was set aside for 12 h. Water was added to precipitate the product, which was collected and dried. The compound was recrystallized from ethanol to yield yellow prisms.

### Refinement

Carbon-bound H atoms were placed in calculated positions (C—H 0.95–0.98 Å) and were included in the refinement in the riding model approximation, with *U*_iso_(H) set to 1.2–1.5 times *U*_eq_(C).

The amino H atoms were located in a difference Fourier map, and were refined isotropically with a distance restraint of N—H 0.88 (1) Å.

The phenylene ring is disordered along the C*_ipso_*—C*_para_* axis; as the disorder refined to nearly 1:1, the ratio was then fixed as exactly 1:1. No restraints were imposed. As the two orientations differ by 60°, the H atoms of the methyl group are ordered. These were refined isotropically with a distance restraint of C—H 0.98 (1) Å.

### Crystal data

**Table:**

C_14_H_15_N_3_O_2_	*Z* = 2
*M**_r_* = 257.29	*F*(000) = 272
Triclinic, *P*1	*D*_x_ = 1.353 Mg m^−^^3^
Hall symbol: -P 1	Mo *K*α radiation, λ = 0.71073 Å
*a* = 5.0521 (6) Å	Cell parameters from 1604 reflections
*b* = 10.4068 (13) Å	θ = 3.0–27.9°
*c* = 12.6558 (16) Å	µ = 0.09 mm^−^^1^
α = 103.295 (2)°	*T* = 100 K
β = 95.338 (2)°	Prism, yellow
γ = 100.072 (2)°	0.30 × 0.06 × 0.03 mm
*V* = 631.39 (13) Å^3^	

### Data collection

**Table:**

Bruker SMART APEX diffractometer	2002 reflections with *I* > 2σ(*I*)
Radiation source: fine-focus sealed tube	*R*_int_ = 0.030
graphite	θ_max_ = 27.5°, θ_min_ = 1.7°
ω scans	*h* = −6→6
6045 measured reflections	*k* = −13→13
2873 independent reflections	*l* = −16→15

### Refinement

**Table:**

Refinement on *F*^2^	Primary atom site location: structure-invariant direct methods
Least-squares matrix: full	Secondary atom site location: difference Fourier map
*R*[*F*^2^ > 2σ(*F*^2^)] = 0.050	Hydrogen site location: inferred from neighbouring sites
*wR*(*F*^2^) = 0.152	H atoms treated by a mixture of independent and constrained refinement
*S* = 1.03	*w* = 1/[σ^2^(*F*_o_^2^) + (0.0719*P*)^2^ + 0.3595*P*] where *P* = (*F*_o_^2^ + 2*F*_c_^2^)/3
2873 reflections	(Δ/σ)_max_ = 0.001
230 parameters	Δρ_max_ = 0.29 e Å^−^^3^
5 restraints	Δρ_min_ = −0.34 e Å^−^^3^

### Special details

**Table:**

Geometry. All e.s.d.'s (except the e.s.d. in the dihedral angle between two l.s. planes) are estimated using the full covariance matrix. The cell e.s.d.'s are taken into account individually in the estimation of e.s.d.'s in distances, angles and torsion angles; correlations between e.s.d.'s in cell parameters are only used when they are defined by crystal symmetry. An approximate (isotropic) treatment of cell e.s.d.'s is used for estimating e.s.d.'s involving l.s. planes.

### Fractional atomic coordinates and isotropic or equivalent isotropic displacement parameters (Å^2^)

**Table:**

	*x*	*y*	*z*	*U*_iso_*/*U*_eq_	Occ. (<1)
O1	−0.1305 (3)	0.14044 (15)	0.52867 (12)	0.0268 (4)	
O2	0.6699 (3)	0.4493 (2)	0.61411 (15)	0.0452 (5)	
N1	0.4515 (3)	0.23144 (16)	0.84070 (13)	0.0174 (4)	
N2	0.2167 (3)	0.15038 (17)	0.78625 (14)	0.0184 (4)	
N3	0.2489 (4)	0.3669 (2)	0.52691 (17)	0.0345 (5)	
C1	0.5330 (4)	0.23207 (19)	0.95263 (16)	0.0172 (4)	
C2	0.5929 (9)	0.1193 (4)	0.9788 (3)	0.0224 (9)	0.50
H2	0.5835	0.0389	0.9235	0.027*	0.50
C3	0.6675 (9)	0.1253 (4)	1.0878 (4)	0.0255 (10)	0.50
H3	0.7061	0.0467	1.1069	0.031*	0.50
C4	0.6885 (4)	0.2436 (2)	1.17193 (17)	0.0232 (5)	
C5	0.6121 (8)	0.3527 (4)	1.1393 (3)	0.0199 (8)	0.50
H5	0.6103	0.4322	1.1939	0.024*	0.50
C6	0.5386 (8)	0.3491 (4)	1.0303 (3)	0.0210 (8)	0.50
H6	0.4933	0.4257	1.0098	0.025*	0.50
C7	0.7753 (5)	0.2541 (3)	1.2913 (2)	0.0349 (6)	
H7A	0.643 (6)	0.288 (4)	1.336 (3)	0.088 (13)*	
H7B	0.958 (3)	0.309 (3)	1.312 (3)	0.064 (10)*	
H7C	0.782 (6)	0.1647 (16)	1.302 (3)	0.058 (9)*	
C8	0.8281 (4)	0.4154 (2)	0.82810 (17)	0.0206 (4)	
H8A	0.9078	0.3966	0.8951	0.031*	
H8B	0.7935	0.5072	0.8451	0.031*	
H8C	0.9542	0.4070	0.7737	0.031*	
C9	0.5672 (4)	0.31710 (19)	0.78304 (17)	0.0185 (4)	
C10	0.3949 (4)	0.29140 (19)	0.68544 (16)	0.0179 (4)	
C11	0.1794 (4)	0.18543 (19)	0.69107 (16)	0.0172 (4)	
C12	−0.0704 (4)	0.1115 (2)	0.61502 (17)	0.0187 (4)	
C13	−0.2497 (4)	0.0000 (2)	0.64740 (18)	0.0219 (5)	
H13A	−0.4213	−0.0288	0.5975	0.033*	
H13B	−0.2862	0.0325	0.7226	0.033*	
H13C	−0.1586	−0.0764	0.6432	0.033*	
C14	0.4481 (4)	0.3732 (2)	0.60424 (17)	0.0201 (4)	
C2'	0.7886 (8)	0.2071 (4)	0.9862 (4)	0.0223 (9)	0.50
H2'	0.9071	0.1855	0.9337	0.027*	0.50
C3'	0.8673 (8)	0.2140 (4)	1.0949 (4)	0.0245 (9)	0.50
H3'	1.0412	0.1989	1.1178	0.029*	0.50
C5'	0.4431 (9)	0.2659 (5)	1.1399 (4)	0.0268 (10)	0.50
H5'	0.3240	0.2862	1.1923	0.032*	0.50
C6'	0.3625 (9)	0.2593 (4)	1.0304 (3)	0.0226 (9)	0.50
H6'	0.1872	0.2738	1.0088	0.027*	0.50
H31	0.093 (4)	0.311 (3)	0.522 (3)	0.059 (9)*	
H32	0.280 (6)	0.422 (2)	0.484 (2)	0.051 (9)*	

### Atomic displacement parameters (Å^2^)

**Table:**

	*U*^11^	*U*^22^	*U*^33^	*U*^12^	*U*^13^	*U*^23^
O1	0.0255 (8)	0.0296 (8)	0.0230 (8)	−0.0029 (6)	−0.0035 (6)	0.0114 (6)
O2	0.0267 (9)	0.0648 (13)	0.0474 (12)	−0.0126 (8)	−0.0049 (8)	0.0411 (10)
N1	0.0167 (8)	0.0169 (8)	0.0174 (9)	0.0004 (6)	−0.0008 (6)	0.0054 (7)
N2	0.0151 (8)	0.0192 (8)	0.0191 (9)	0.0005 (6)	−0.0009 (7)	0.0048 (7)
N3	0.0266 (10)	0.0430 (13)	0.0344 (12)	−0.0089 (9)	−0.0065 (9)	0.0266 (10)
C1	0.0174 (9)	0.0174 (9)	0.0161 (10)	0.0000 (7)	−0.0006 (7)	0.0063 (8)
C2	0.027 (2)	0.0164 (19)	0.019 (2)	−0.0017 (16)	−0.0016 (17)	0.0004 (16)
C3	0.034 (2)	0.018 (2)	0.025 (2)	−0.0007 (17)	−0.0039 (18)	0.0129 (17)
C4	0.0245 (10)	0.0233 (11)	0.0194 (11)	−0.0038 (8)	−0.0009 (8)	0.0087 (8)
C5	0.0180 (19)	0.022 (2)	0.017 (2)	0.0020 (16)	0.0031 (15)	0.0010 (16)
C6	0.0204 (19)	0.023 (2)	0.020 (2)	0.0039 (16)	0.0012 (16)	0.0069 (16)
C7	0.0383 (14)	0.0406 (15)	0.0241 (13)	−0.0015 (11)	−0.0047 (11)	0.0154 (11)
C8	0.0189 (10)	0.0205 (10)	0.0220 (11)	0.0012 (8)	0.0013 (8)	0.0072 (8)
C9	0.0189 (10)	0.0161 (9)	0.0211 (10)	0.0039 (8)	0.0038 (8)	0.0053 (8)
C10	0.0187 (9)	0.0174 (10)	0.0175 (10)	0.0036 (8)	0.0033 (8)	0.0039 (8)
C11	0.0169 (9)	0.0164 (9)	0.0186 (10)	0.0037 (7)	0.0031 (8)	0.0043 (8)
C12	0.0175 (9)	0.0183 (10)	0.0204 (11)	0.0043 (8)	0.0032 (8)	0.0043 (8)
C13	0.0200 (10)	0.0219 (10)	0.0221 (11)	−0.0017 (8)	0.0005 (8)	0.0076 (8)
C14	0.0207 (10)	0.0190 (10)	0.0213 (11)	0.0033 (8)	0.0040 (8)	0.0067 (8)
C2'	0.0161 (19)	0.026 (2)	0.026 (2)	0.0033 (16)	0.0012 (16)	0.0100 (17)
C3'	0.0155 (19)	0.029 (2)	0.028 (2)	−0.0003 (16)	−0.0057 (17)	0.0122 (18)
C5'	0.033 (2)	0.026 (2)	0.020 (2)	0.0043 (19)	0.0007 (18)	0.0058 (18)
C6'	0.026 (2)	0.021 (2)	0.022 (2)	0.0069 (17)	−0.0035 (17)	0.0092 (17)

### Geometric parameters (Å, °)

**Table:**

O1—C12	1.224 (2)	C6—H6	0.9500
O2—C14	1.232 (3)	C7—H7A	0.980 (10)
N1—N2	1.351 (2)	C7—H7B	0.975 (10)
N1—C9	1.366 (3)	C7—H7C	0.977 (10)
N1—C1	1.436 (3)	C8—C9	1.493 (3)
N2—C11	1.343 (3)	C8—H8A	0.9800
N3—C14	1.319 (3)	C8—H8B	0.9800
N3—H31	0.88 (3)	C8—H8C	0.9800
N3—H32	0.89 (3)	C9—C10	1.386 (3)
C1—C2	1.365 (5)	C10—C11	1.427 (3)
C1—C6	1.372 (5)	C10—C14	1.492 (3)
C1—C6'	1.381 (5)	C11—C12	1.485 (3)
C1—C2'	1.408 (4)	C12—C13	1.502 (3)
C2—C3	1.380 (6)	C13—H13A	0.9800
C2—H2	0.9500	C13—H13B	0.9800
C3—C4	1.412 (5)	C13—H13C	0.9800
C3—H3	0.9500	C2'—C3'	1.379 (6)
C4—C5'	1.344 (5)	C2'—H2'	0.9500
C4—C5	1.395 (5)	C3'—H3'	0.9500
C4—C3'	1.408 (5)	C5'—C6'	1.390 (6)
C4—C7	1.507 (3)	C5'—H5'	0.9500
C5—C6	1.386 (6)	C6'—H6'	0.9500
C5—H5	0.9500		
			
N2—N1—C9	112.79 (16)	H7B—C7—H7C	108 (3)
N2—N1—C1	119.30 (15)	C9—C8—H8A	109.5
C9—N1—C1	127.38 (16)	C9—C8—H8B	109.5
C11—N2—N1	105.11 (15)	H8A—C8—H8B	109.5
C14—N3—H31	119 (2)	C9—C8—H8C	109.5
C14—N3—H32	117 (2)	H8A—C8—H8C	109.5
H31—N3—H32	124 (3)	H8B—C8—H8C	109.5
C2—C1—C6	122.6 (3)	N1—C9—C10	106.38 (17)
C2—C1—C6'	96.6 (3)	N1—C9—C8	121.63 (18)
C6—C1—C6'	50.4 (3)	C10—C9—C8	131.98 (18)
C2—C1—C2'	51.4 (3)	C9—C10—C11	104.86 (17)
C6—C1—C2'	99.4 (3)	C9—C10—C14	121.36 (17)
C6'—C1—C2'	118.4 (3)	C11—C10—C14	133.68 (18)
C2—C1—N1	120.7 (2)	N2—C11—C10	110.85 (17)
C6—C1—N1	116.7 (2)	N2—C11—C12	116.22 (17)
C6'—C1—N1	120.4 (2)	C10—C11—C12	132.92 (18)
C2'—C1—N1	121.1 (2)	O1—C12—C11	121.39 (18)
C1—C2—C3	118.2 (4)	O1—C12—C13	120.63 (18)
C1—C2—H2	120.9	C11—C12—C13	117.98 (18)
C3—C2—H2	120.9	C12—C13—H13A	109.5
C2—C3—C4	122.5 (4)	C12—C13—H13B	109.5
C2—C3—H3	118.8	H13A—C13—H13B	109.5
C4—C3—H3	118.8	C12—C13—H13C	109.5
C5'—C4—C3'	120.3 (3)	H13A—C13—H13C	109.5
C5—C4—C3	115.9 (3)	H13B—C13—H13C	109.5
C5'—C4—C7	119.8 (3)	O2—C14—N3	121.8 (2)
C5—C4—C7	120.3 (3)	O2—C14—C10	119.67 (19)
C3'—C4—C7	119.9 (3)	N3—C14—C10	118.44 (18)
C3—C4—C7	123.7 (3)	C3'—C2'—C1	120.2 (4)
C6—C5—C4	122.4 (4)	C3'—C2'—H2'	119.9
C6—C5—H5	118.8	C1—C2'—H2'	119.9
C4—C5—H5	118.8	C2'—C3'—C4	119.6 (4)
C1—C6—C5	118.3 (4)	C2'—C3'—H3'	120.2
C1—C6—H6	120.9	C4—C3'—H3'	120.2
C5—C6—H6	120.9	C4—C5'—C6'	120.4 (4)
C4—C7—H7A	111 (2)	C4—C5'—H5'	119.8
C4—C7—H7B	108 (2)	C6'—C5'—H5'	119.8
H7A—C7—H7B	114 (3)	C1—C6'—C5'	121.1 (4)
C4—C7—H7C	109.8 (19)	C1—C6'—H6'	119.5
H7A—C7—H7C	106 (3)	C5'—C6'—H6'	119.5
			
C9—N1—N2—C11	−0.3 (2)	N1—C9—C10—C14	176.00 (18)
C1—N1—N2—C11	171.92 (17)	C8—C9—C10—C14	−3.1 (3)
N2—N1—C1—C2	64.4 (3)	N1—N2—C11—C10	−0.3 (2)
C9—N1—C1—C2	−124.6 (3)	N1—N2—C11—C12	−179.27 (16)
N2—N1—C1—C6	−113.7 (3)	C9—C10—C11—N2	0.7 (2)
C9—N1—C1—C6	57.3 (3)	C14—C10—C11—N2	−175.6 (2)
N2—N1—C1—C6'	−55.8 (3)	C9—C10—C11—C12	179.5 (2)
C9—N1—C1—C6'	115.2 (3)	C14—C10—C11—C12	3.2 (4)
N2—N1—C1—C2'	125.1 (3)	N2—C11—C12—O1	176.23 (19)
C9—N1—C1—C2'	−63.8 (3)	C10—C11—C12—O1	−2.5 (3)
C6—C1—C2—C3	−1.4 (5)	N2—C11—C12—C13	−3.4 (3)
C6'—C1—C2—C3	−48.0 (4)	C10—C11—C12—C13	177.8 (2)
C2'—C1—C2—C3	73.4 (4)	C9—C10—C14—O2	12.5 (3)
N1—C1—C2—C3	−179.4 (3)	C11—C10—C14—O2	−171.7 (2)
C1—C2—C3—C4	−1.2 (6)	C9—C10—C14—N3	−163.9 (2)
C2—C3—C4—C5'	50.2 (5)	C11—C10—C14—N3	12.0 (4)
C2—C3—C4—C5	4.0 (5)	C2—C1—C2'—C3'	−76.6 (4)
C2—C3—C4—C3'	−75.0 (4)	C6—C1—C2'—C3'	48.0 (4)
C2—C3—C4—C7	−178.6 (3)	C6'—C1—C2'—C3'	−1.9 (5)
C5'—C4—C5—C6	−77.4 (4)	N1—C1—C2'—C3'	177.2 (3)
C3'—C4—C5—C6	46.3 (4)	C1—C2'—C3'—C4	1.2 (6)
C3—C4—C5—C6	−4.5 (5)	C5'—C4—C3'—C2'	−0.4 (5)
C7—C4—C5—C6	178.0 (3)	C5—C4—C3'—C2'	−46.8 (4)
C2—C1—C6—C5	1.0 (5)	C3—C4—C3'—C2'	70.1 (4)
C6'—C1—C6—C5	70.3 (4)	C7—C4—C3'—C2'	−178.8 (3)
C2'—C1—C6—C5	−48.9 (4)	C5—C4—C5'—C6'	72.9 (4)
N1—C1—C6—C5	179.0 (3)	C3'—C4—C5'—C6'	0.3 (6)
C4—C5—C6—C1	2.2 (6)	C3—C4—C5'—C6'	−47.5 (4)
N2—N1—C9—C10	0.8 (2)	C7—C4—C5'—C6'	178.7 (3)
C1—N1—C9—C10	−170.69 (18)	C2—C1—C6'—C5'	51.2 (4)
N2—N1—C9—C8	−179.98 (17)	C6—C1—C6'—C5'	−76.3 (4)
C1—N1—C9—C8	8.5 (3)	C2'—C1—C6'—C5'	1.8 (5)
N1—C9—C10—C11	−0.9 (2)	N1—C1—C6'—C5'	−177.3 (3)
C8—C9—C10—C11	−180.0 (2)	C4—C5'—C6'—C1	−1.0 (6)

### Hydrogen-bond geometry (Å, °)

**Table:**

*D*—H···*A*	*D*—H	H···*A*	*D*···*A*	*D*—H···*A*
N3—H31···O1	0.88 (3)	1.95 (2)	2.771 (2)	154 (3)
N3—H32···O2^i^	0.89 (3)	2.02 (1)	2.906 (3)	177 (3)

Symmetry codes: (i) −*x*+1, −*y*+1, −*z*+1.
